# The Strange Case of Jekyll and Hyde: Parallels Between Neural Stem Cells and Glioblastoma-Initiating Cells

**DOI:** 10.3389/fonc.2020.603738

**Published:** 2021-01-08

**Authors:** David Bakhshinyan, Neil Savage, Sabra Khalid Salim, Chitra Venugopal, Sheila K. Singh

**Affiliations:** ^1^Department of Biochemistry and Biomedical Sciences, Faculty of Health Sciences, McMaster University, Hamilton, ON, Canada; ^2^Department of Surgery, Faculty of Health Sciences, McMaster University, Hamilton, ON, Canada

**Keywords:** glioblastoma stem cells, neural stem cells, neurogenic niche, tumor microenvironment, tumor metabolism

## Abstract

During embryonic development, radial glial precursor cells give rise to neural lineages, and a small proportion persist in the adult mammalian brain to contribute to long-term neuroplasticity. Neural stem cells (NSCs) reside in two neurogenic niches of the adult brain, the hippocampus and the subventricular zone (SVZ). NSCs in the SVZ are endowed with the defining stem cell properties of self-renewal and multipotent differentiation, which are maintained by intrinsic cellular programs, and extrinsic cellular and niche-specific interactions. In glioblastoma, the most aggressive primary malignant brain cancer, a subpopulation of cells termed glioblastoma stem cells (GSCs) exhibit similar stem-like properties. While there is an extensive overlap between NSCs and GSCs in function, distinct genetic profiles, transcriptional programs, and external environmental cues influence their divergent behavior. This review highlights the similarities and differences between GSCs and SVZ NSCs in terms of their gene expression, regulatory molecular pathways, niche organization, metabolic programs, and current therapies designed to exploit these differences.

## Introduction

Glioblastoma [GBM, International Classification of Diseases for Oncology (ICD-O) code 9440/3] is the most common and aggressive primary CNS malignancy in adults. The short median survival of 9–18 months in patients with GBM has been attributed to the highly invasive nature of the disease with rapid cell infiltration, frequent relapses, and therapy resistance (1–5). Anatomically, GBMs arise predominantly in the cerebral cortex (40%), followed by temporal lobe (29%), the parietal lobe (14%), deeper brain structures (14%), and the occipital lobe (3%) (6). The current therapy for GBM, consisting of maximal surgical resection followed by radiation and temozolomide (TMZ), a cytotoxic chemotherapy (7), has yielded minimal survival benefit with a vast majority of GBM patients presenting with tumor recurrence. Recently, the addition of tumor-treating fields (TTFs) to the standard chemoradiotherapy regimen has extended survival of patients from 16 to 20.9 months (8).

Limited therapeutic options, poor survival, and the universally fatal nature of the disease have fueled research efforts to uncover novel molecular vulnerabilities within GBM. However, despite the best efforts, the discovery of novel effective treatments remains elusive. GBM tumors exhibit a large degree of intra- and inter-tumoral heterogeneity, which frequently renders majority of targeted therapies ineffective (9). In an attempt to deconvolute this heterogeneity, an increasing body of scientific work has been established to identify the cell-of-origin in GBM to shed light on the hierarchical organization of GBM tumors and identify vulnerabilities to target the tumor at its roots. Historically, two major candidate cells of origin of GBMs have been proposed, neural stem cells (NSCs) and oligodendrocyte precursor cells (OPCs). The supporting evidence and shortcomings of the two hypotheses have been recently reviewed in detail by Fan et al. (10). The initial identification of a subpopulation of GBM cells with multilineage potency, increased self-renewal ability, proliferation, and migration, termed glioma stem cells (GSCs) (11–13) has provided correlative evidence for the possibility of GBMs arising from transformed neural stem cells (NSCs). Through analysis of patient samples and genetically engineered mouse models of GBM, several studies have subsequently provided molecular evidence suggesting that GBM arises from migration of mutated, astrocyte-like NSCs from the subventricular zone (SVZ) (11–15). In this review, we describe the intrinsic and extrinsic regulations of SVZ NSCs and GSCs including molecular pathways, microenvironment, and metabolic activity to further evaluate how the differences can be exploited in the next generation of targeted therapies for GBM.

## Subventricular Zone Neural Stem Cells in Adult Neurogenesis

Neural stem and progenitor cells are a specialized population of multipotent cells that contribute to lifelong neural plasticity. During embryonic neurogenesis, NSCs are spatiotemporally regulated to generate multiple neural populations including neurons and glial cells (16). Beyond development, a small pool of NSCs are maintained and become spatially restricted to two neurogenic niches in the brain; the dentate gyrus of the hippocampus known as the subgranular zone (SGZ), or the ventricular-subventricular zone (SVZ) (17). NSCs were long believed to be a retained pool of self-renewing stem cells as suggested by long-term expansion and retention of differentiation potential by neurosphere culturing (17). Much of our current understanding of the human SVZ has been derived from studies in other mammals, namely mice. While mice display robust SVZ neurogenesis, humans have shown an increased preponderance of SGZ or hippocampal neurogenesis (18). Nonetheless, studies in other mammals provide deep insight into comparable NSC regulation and differentiation, highlighting significant complexity and heterogeneity in the adult brain. The SVZ is the largest germinal center in the adult human brain found on the walls of the lateral ventricles (19). SVZ-NSCs, also known as B1 cells, are displaced and surrounded by bi- or multi-ciliated ependymal cells to form a pinwheel-like structure, in which the NSC apical surface contacts the cerebrospinal fluid (CSF) and some the ventricle, while the basal process terminates vascular vessels and the extravascular basal lamina (17, 20). The morphology of B1 cells is reminiscent of radial glia in the embryonic ventricular zone from which they are hypothesized to originate (20, 21). These NSCs, which express GFAP and CD133 at quiescence, can become activated, express Nestin and EGFR, and become highly proliferative (22). Activation of these NSCs ultimately gives rise to EGFR+ transient amplifying cells, which in turn differentiate into progenitors and finally, neuroblasts. These cells follow a specialized migratory route known as the rostral migratory stream to the olfactory bulb in which they disperse radially and differentiate into GABAergic interneurons, or form corpus callosum oligodendrocytes (23, 24). Purification and subsequent single-cell transcriptomics have revealed that SVZ-NSCs exhibit a phenotypic continuum between quiescence and activation suggesting a high degree of transcriptional dynamics (21, 25, 26). NSCs present a heterogeneous profile of multiple activation states in the adult SVZ niche regulated by various molecular programs affected by both intrinsic and extrinsic programs (21, 23).

### Intrinsic Regulation

Adult NSC self-renewal and multipotency have been proposed to be regulated by various transcriptional factors. One such factor is the orphan nuclear receptor TLX, which has been shown to be an essential transcriptional regulator of NSC maintenance and proliferation in the adult brain (27). Transcriptional regulation has also been demonstrated to be controlled by arsenite-resistance protein 2, a critical activator of the Sox family of DNA binding proteins, particularly Sox2 (28). TLX has also been suggested to regulate NSC maintenance by repression of cell-cycle inhibitory factors and recruitment of a host of tumor suppressor genes including Bmi1 (29), p53 (30), and the PTEN pathway (31) which regulate stem cell maintenance (27). Adult SVZ NSCs have also been shown to be regulated by basic helix-loop-helix (bHLH) transcription factors, which inhibit differentiation and maintain stemness. BHLH genes, particularly of the Hes family have also been implicated as Notch signaling effectors, which inhibit neuronal differentiation, and maintain NSCs by inducing quiescence (17, 32, 33). Beyond transcriptional regulators, other nuclear receptors such as estrogen receptors (34), thyroid hormone receptors (35), and peroxisome proliferator activated receptor-gamma (36), have been shown to regulate NSC proliferation and differentiation (17, 32).

Cell-intrinsic regulation is also maintained through epigenetic modification and chromatin remodeling. Epigenetic control has been demonstrated to be regulated by the aforementioned polycomb repressor Bmi1 by methylation of the histone tail H3K27 to promote self-renewal (37). SVZ-NSC differentiation is alternatively regulated by methylation of the histone tail H3K4 by the TrxG family of proteins (38). The balance between self-renewal and differentiation is subsequently mediated by switches from a polycomb-repressor driven chromatin remodeling to that of the TrxG family (39, 40). Epigenetic regulation has also been shown to work through histone acetyltransferase (HATs) and deacetylases (HDACs) in NSCs (41). HDACs promote the silencing of key neurogenic transcription and cell-cycle factors in a comparatively more dynamic fashion relative to the polycomb family of epigenetic regulators to tightly regulate fate specification, differentiation, and cell-cycle exit (32, 37).

Epigenetic mechanisms in SVZ-NSCs are also regulated by a network of miRNAs and non-coding RNAs, which play an additional regulatory role in adult neurogenesis. Many members of the small RNA family have been implicated in modulating neuronal differentiation by binding to the RE1-silencing transcription factor (REST), a crucial regulator of neuronal gene expression (42). Together, small non-coding RNAs fine tune epigenetic programs to regulate cell states of SVZ-NSCs.

### Extrinsic Regulation

NSC intrinsic programs are also regulated by signals from the neurogenic niche (37, 43). The NSC niche is an extensive microenvironment that hosts cell-cell and cell-microenvironment interactions (44). Here, NSC proliferation and fate determination is facilitated by various cell-extrinsic molecular signals.

**Figure 1:**
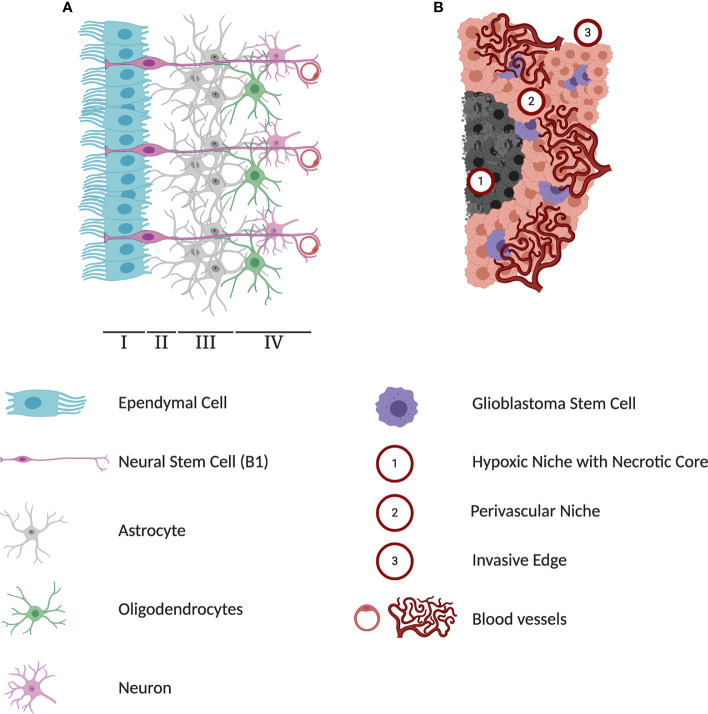
Differences in cytoarchitectures surrounding subventricular zone (SVZ) neural stem cells (NSCs) and glioblastoma stem cells (GSCs). **(A)** A schematic representation of adult human SVZ. Human SVZ is composed of four distinct layers. The superficial ependymal monolayer, Lamina I, is in contact with ventricular lumen. The second layer, Lamina II is a vastly acellular layer formed by the neuroblast depletion, containing ependymal expansions and numerous astrocyte processes. Lamina III, is a region known as astrocytic ribbon, containing densely packed astrocytes. Lastly, Lamina IV is a transitional zone rich in oligodendrocytes and myelinated neurons. The NSC niche is an extensive microenvironment that hosts cell-cell and cell-microenvironment interactions that contribute extensively to the extrinsic regulation of NSC proliferation and self-renewal. **(B)** A schematic representation of three major microenvironments within glioblastoma (GBM) tumors. The hypoxia region formed in the course of tumor growth lacks any blood and oxygen supply and has been implicated in playing a protective role against chemoradiotherapy. Perivascular niches exist along capillaries or arterioles where endothelial cells come into direct contact with glioblastoma cells. In addition to producing high levels of pro-angiogenic factors driving tumor vascularization, cells in the perivascular contribute to activation of pathways regulating self-renewal and proliferation of GSCs. As a highly invasive tumor, GBMs can infiltrate into healthy brain tissue and limit the effectiveness of surgical interventions.

The extracellular matrix is a critical component of the SVZ niche that has been identified as a regulator of NSC proliferation (**Figure 1A**). It is composed of vessel basal lamina rich in laminin, collagen-I, and other molecules including metalloproteinases, brevican, tenascin-C, growth factors, and proteoglycans (44, 45). Unlike embryonic development, the adult SVZ also consists of a unique extravascular component consisting of ECM aggregates near the ventricular surface known as fractones (46). Fractones play an important role in facilitating the binding of growth-factors, cytokines, and chemokines from the circulating CSF to fractone-associated heparan sulfate proteoglycans (HSPGs) (47). Fractones then present these molecules to their cognate receptors on NSCs. Some of these molecules include fibroblast growth factor-2 (FGF-2) (48) and bone morphogenetic proteins (BMP) to influence NSC proliferation (49).

Molecular signals in the ECM regulating adult NSC activity can originate from multiple cell types. In assessing the cellular composition of the SVZ microenvironment, endothelial, pericyte and vascular cells, as well as immature and mature lineages of NSCs are found (50, 51). Most significant are the ependymal cells which uniquely line the ventricular surface in a pinwheel formation around single NSCs (20, 52). Ependymal cells secrete local signaling factors into the circulating CSF, which include noggin, a BMP signaling inhibitor, to activate adult human NSCs and promote fate commitment (53). Endothelial cells in the SVZ also secrete factors including vascular endothelial growth factor (VEGF) and neurotrophin-3 which promote self-renewal and quiescence, respectively (54). NSCs and their immediate progeny also self-regulate through autocrine and paracrine mechanisms (55, 56). This regulation is particularly controlled by diffusible factors transmitted through gap junctions such as the neurotransmitter GABA which modulates quiescence (57), and cytokines such as IL-1β and IL-6 which promote NSC differentiation (58). Cell-cell interactions of adult NSCs are also observed in the SVZ microenvironment through signaling molecules such as ephrin B2 and Jagged1 on endothelial cells which promote quiescence and cell cycle suppression (59). Direct cell-cell interactions have also been demonstrated, such as that with endothelial cells through α6β1 integrins which modulate NSC proliferation (60). Thus, multiple cell types contribute to the signaling *milieu* of the SVZ niche.

Extrinsic factors that affect adult NSC regulation can also be derived from the cerebrospinal fluid (CSF) and blood-derived systemic signals. NSCs have direct access to the CSF which provides a rich supply of various additional mitogens such as PDGF, and morphogens including the Wnt ligands which particularly promote proliferation and self-renewal of adult NSCs *via* canonical Wnt signaling (61). Peripheral circulating morphogens have also been implicated in modulating mouse NSC behavior such as GDF11 which induces vascular remodeling leading to NSC proliferation (62, 63).

NSC multipotency and stemness is also modified by endogenous and niche-derived metabolic factors. Adult NSCs are known to rely on aerobic glycolysis prior to differentiation. Changes in metabolic activity affects adult NSC differentiation and cell-fate commitment, particularly by activation of mitochondrial respiration and reactive oxygen species production (64). Oxygen tension or hypoxia in the microenvironment also stimulates proliferation within the SVZ and migration into the hypoxic region (65). Metabolomic analyses of NSCs has also revealed that lipid metabolism can induce changes in NSC state. Adult NSCs have been shown to require lipogenesis for proliferation to ensure quiescence (66, 67). Extrinsic insulin/insulin-like growth factor signaling has also been shown to stimulate NSC reactivation and proliferation through regulation of CDK4 activity (68, 69).

While other niche-mediated cues such as regional identity (70) and positional information (71) modulate adult NSC activity, it is the combination of molecular stimuli, cytoarchitecture, and structural components of the SVZ niche that continually regulate NSC state and function.

## Cancer Stem Cell Hypothesis and Glioblastoma

The cancer stem cell (CSC) hypothesis has been used as a framework describe and provide explanation for the high degree of molecular heterogeneity, cellular plasticity, and the molecular divergence of recurrent GBM. CSCs were observed to share many of the similar properties to the healthy stem cells including multipotent differentiation and self-renewal (72), low frequency and low proliferative rate (73–76), ability to regulate the surrounding microenvironment (77), strict re-regulation of proliferation and cell death, and reliance of similar molecular pathways (78). The initial evidence of cancer stem cell-driven tumorigenesis came through studies involving serial re-transplantation of a specific subpopulation of leukemic cells in immunodeficient mice (74, 79). Since the early 2000s, CSCs have successfully been identified in numerous solid tumors including breast cancer (80), colorectal cancer (81, 82), and brain cancers including GBM (12) in which they are specifically termed GBM stem cells (GSCs). GSCs have demonstrated chemo- (83, 84) and radiotherapy (85) resistant, while contributing to invasion (86), angiogenesis (87) and tumor recurrence (87). Comparison of underlying molecular mechanisms within GSCs to those in NSCs will allow for development of selective therapies to target the rare cell population responsible for tumor initiation, propagation, and evasion of current therapies.

While the precise identification of the GBM cell of origin remains elusive, two major hypotheses have been explored over the years. In one theory, GBM arises from a transformation events in differentiated astrocytes, while others have suggested that a GBM pathogenesis begins with a transformed NSC [Comprehensive review by Fan et al. (10)]. Previously, astrocyte progenitor cells were believed to be the sole proliferating cells in the adult brain (88) and were hypothesized to drive GBM tumorigenesis due to extensive expression of the marker GFAP in both healthy astrocytes and glioma samples (89). This would require a fully committed astrocytes to acquire mutations, de-differentiate and become tumorigenic. Other attempts to identify the cell-of-origin in GBM using lineage tracing experiments in mouse models have suggested oligodendrocyte precursor cells (OPCs) (90, 91). The similarity in expression levels of PDGFRα and NG2 in OPCs and GBM provided further support to the notion of OPC-derived GSC (92–94), and in a study by Hide et al., the authors have proposed a model where a transformation of both OPCs and NSCs is required for generation of GSCs (90).

Early mouse models exploring effects of genetic alterations in either NSCs or differentiated astrocytes have failed to provide definitive resolution to the cell-of-origin question. Some reports suggested that overexpression of Ras and Akt signaling in neural progenitor cells but not in more differentiated astrocytes was sufficient to induce formation of GBM-like lesions (95). On the other hand, other groups have provided evidence that genetic alterations in either population is sufficient to induce GBM formation (96). Mounting evidence of the hierarchical organization of GBM tumors and the upregulation of developmental pathways in GSCs became the principal evidence for the notion of transformation of NSCs from SVZ as the initial stage of gliomagenesis. In addition to evident functional overlap, similarities in expression patterns of a number of genes including CD133 (97, 98), Sox10 (99), Nestin (100, 101), Musashi (101, 102), GFAP (103), and Olig1/2 (104, 105) highlight shared molecular programs between NSCs and GSCs. Through deep sequencing of isocitrate dehydrogenase wild-type GBM patient samples and normal SVZ tissue, researchers observed similar expression of driver mutations in both the SVZ and patient matched-tumor tissue (15). Intriguingly, multiple studies have reported shorter survival of GBM patients in cases where tumors were in contact with SVZ (106–109). The comprehensive profiling and understanding of GBM cell of origin may pave the way for identification of prognostic markers along with targeted preventative and curative therapies.

## Intrinsic Deregulations of Glioma Stem Cells Compared to Neural Stem Cells

**Figure 2:**
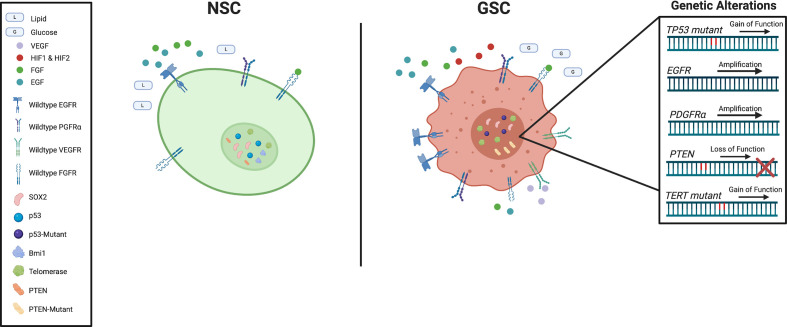
Schematic representation of key genetic and signaling difference between subventricular zone (SVZ) neural stem cells (NSCs) and glioblastoma stem cells (GSCs).

Like all cancers, GBM exhibits behavioral hallmarks that distinguish it from healthy tissue (110). Compared to NSCs, GSCs are self-sufficient in providing growth signals, resistant to growth inhibition, evade programmed cell death, have limitless replicative potential, sustain angiogenesis, and invade surrounding tissue (**Figure 2**). Markers of interest to explain these phenotypes have been extensively studied and while some have been exploited in clinical settings, no single one is responsible for GBM’s relentless growth.

### Aberrant Growth Signals

Epidermal growth factor (EGF), is a critical regulator in the proliferation of normal NSCs in mice (111). Aberrant EGF signaling prevents mouse NSC differentiation while increasing proliferative capacity and invasiveness, properties that closely resemble those of high-grade gliomas (112, 113). As epidermal growth factor receptor (*EGFR*) amplification is one of the most frequent mutations in GBM patients (114), and has been implicated in human gliomagenesis (115), its targeting in the clinical setting has been extensively investigate (116). Even in the absence of EGF, aberrant behavior of the EGFR pathway maintains stemness properties and promotes self-sufficient growth in tumors human (117).

Along with EGF, fibroblast growth factors (FGF) play an important role in the regulation of stemness in GSCs *in vitro* (118, 119). The FGF superfamily consists of 22 genes with various isoforms (120). Of particular interest is FGF-2, which does not follow conventional secretion (121) and was found to increase proliferation of NSCs in rat SVZs (122). The low molecular weight isoform of FGF-2 can be excreted and internalized for autocrine or paracrine signaling *via* fibroblast growth factor receptors (FGFRs), or be translocated directly to the cytoplasm and nucleus (123). The transcription factor ZEB1, which has previously been implicated in regulation of glioma stemness (124), has been found to regulate FGFR1 expression (125), suggesting that FGFRs could also be associated with GSCs. Indeed, FGFR1 was found to be preferentially expressed on GSCs, and regulated stem cell transcription factors SOX2, OLIG2, and ZEB1 to promote GBM growth *in vivo* (126). While FGF is a large and cumbersome family to investigate, further inquiries into their functions and potential redundancies may offer insights into preferential novel therapeutic vulnerabilities of GSCs compared to NSCs.

Platelet-derived growth factor (PDGF) is another important regulator of NSCs in the SVZ. When neural stem cells were identified to express PDGF-receptor-alpha (PDGFRA) in the adult mouse SVZ, supplemental PDGF alone was sufficient to induce hyperplasia with some features of GBM (127). These receptors show functional significance by promoting evasion of radiotherapy (128). Amplification of *PDGFRA* is one of the most common mutations in GBM and is most commonly associated with the proneural subtype (129). As PDGFRs are important in the healthy development of the normal central nervous system (130), its increased activity has led to important relationships between subclasses of GBM (131). While these receptors provide another intriguing avenue to potentially eradicate GBM, selective inhibition to avoid targeting of surrounding healthy tissue requires further research and clinical investigation.

### Insensitive to Growth Inhibition

As one of the most studied tumor suppressors in cancer, TP53 has been shown to regulate a wide range of cellular functions of various cancers including GBM. As a key stem cell maintenance regulator, TP53 is expressed in proliferating cells within the SVZ and has been implicated in controlling cell division and differentiation (132). The key regulatory role of TP53 is not limited to development as it has been shown to regulate proliferation and self-renewal of NSCs in adult mice (133, 134). While deletion mutations in TP53 are predominant in several cancers, in the context of GBM, mutations in TP53 are often gain-of-function, resulting in a wider range of downstream effect (135). Mutations in TP53 may cause healthy NSCs to prematurely migrate out of the SVZ effectively seeding the brain with pre-cancerous stem cells (136, 137).

Another common loss of tumor suppressor in GBM is phosphatase and tensin homolog (PTEN). The role of PTEN in the regulation of mouse NSCs in SVZ is extensively reviewed in Li et al. (138). Together with TP53, PTEN also regulates the self-renewal and differentiation of both NSCs and GSCs (139). Loss of PTEN was observed to represses GSC differentiation (140), and similarly promote NSC conversion to a GSC-like phenotype (141). Unsurprisingly, mutational status of PTEN positively correlates with a worse overall prognosis for GBM patients (142).

### Evasion of Programmed Cell Death

The PI3K/AKT/mTOR intracellular pathway is a critical pathway for cell cycle regulation, proliferation, and it is directly antagonized by PTEN (143). The pathway exerts direct influences on cell quiescence, proliferation, longevity by acting predominately through phosphorylation and subsequent activation of AKT/mTOR driving downstream effects (144). Although the PI3K/AKT/mTOR pathway is found throughout the body, its role in promoting growth and proliferation and preventing differentiation in adult NSCs (145) make it an important area of research in GBM and GSCs (146). In addition to being a key pathway in preventing GSC differentiation (147), PI3K/AKT/mTOR further contributes to GBM growth by blocking apoptosis signaling (146).

Signal transducer and activator of transcription-3 (STAT3) is a transcription factor whose activity is directly regulated by PI3K/AKT/mTOR (148). A variety of cytokines, growth factors and interferons converge to regulate STAT3, but its influence on an array of pathways within normal and cancerous stem cells is well documented as it is highly conserved (149). Most importantly, STAT3 plays a major role in maintaining stemness and promoting tumor survival and invasion while suppressing anti-tumor immunity (150). Several studies have shown that reducing levels of STAT3 can lead to a reduction of CD133 and other stemness markers while increasing the propensity for apoptosis and differentiation (151, 152). Inhibition of STAT3 in recurrent GBM has also been shown to reduce levels of BCL-XL and survivin, leading to caspase-3 activation and apoptosis in GSCs (153).

### Limitless Replicative Potential

Healthy replication of cells requires proper activity of telomerase enzymes to ensure the end of chromosomes do not shorten or fuse with other chromosomes (154). Telomerase activity becomes restricted to the SVZ as mammals age (155) but proper maintenance allows NSCs to remain present into adulthood (156). Likewise, increased activity of telomerase leads to replicative immortality within GSCs and is one of the most frequent mutations in GBM (157, 158). The most common gain-of-function mutation of telomerase is located in the promoter region of *TERT* (159) and is predictive of shorter survival times (160).

### Sustained Angiogenesis

Angiogenesis is a tightly controlled pathways in normal tissue and is initiated in response to injury (161). However, several of the receptors discussed before that are upregulated in GSCs are also involved in angiogenic pathways. EGFR, PDGFR, FGFR, and VEGFR have all been shown be involved in angiogenesis with GBM cells and GSCs themselves being major producers of the signaling molecules and their respective receptors (162–167).

Similar, to the intrinsic regulation seen in NSCs, GSCs themselves can directly promote their own survival by modulating the microenvironment. In a study by Takahashi et al., mice engrafted with OCT3/4 overexpressing GBM cells were observed to have larger tumors and increased number of blood vessels (168). Furthermore, tumor-conditioned media accelerated capillary formation *in vitro* and elevated mRNA levels of VEGF in OCT3/4-overexpressing cells providing additional evidence of tumor cell contributing to angiogenesis (168). GSCs have also been shown to directly secrete VEGF-A in extracellular vesicles (169). Along with their influence on surrounding cells, when GSCs asymmetrically divide to self-renew, the differentiated daughter cell is capable of forming blood vessel structures (170).

### Increased Invasiveness

Although GBM cells rarely metastasize to other organs, they do demonstrate a highly invasive growth pattern. Once a GBM is established, the infiltrative edge presents a challenge for surgical resection as the edge is enriched with chemoradioresistant GSCs, meaning the remaining cells are poised to drive tumor relapse upon removal of therapeutic pressures (171, 172). The invasive nature of GBM cells is further exuberated by surrounding non-malignant cells, such as astrocytes secreting cytokines and chemokines (173). Additionally, GBM tumors can degrade the extracellular matrix *via* metalloproteinases (174) and cathepsins (175) to modulate their cell structure for efficient cell movement (176).

### Altered Cellular Metabolism

Compared to the rest of the body, the brain naturally has a higher dependence on glucose as a source of energy, consuming 60% of our daily intake (177). In normal cells, catabolism *via* glycolysis/oxidative phosphorylation and anabolism *via* gluconeogenesis pathways achieve a glucose homeostasis. However, a phenomenon known as the Warburg effect describing the preferential usage of anaerobic glycolysis in CSCs, even in the presence of sufficient oxygen, heightens the dependency of differentiated GBM cells on glucose (178). However, in a side by side comparison, GSCs consumed less glucose and produced less lactate while maintaining higher ATP levels than their differentiated counterparts (179). GSCs are therefore thought to rely mainly on oxidative phosphorylation, however, if challenged, are capable of using other metabolic pathways (179).

Metabolic flexibility and plasticity of cellular states influence each other. In GBM cells, functional p53 leads to increased glutaminase 2 (GLS2) under stress which increases oxidative metabolism and ATP generation, by catalyzing the conversion of glutamine to glutamate and increasing α-ketoglutarate (α-KG) levels (180). This metabolic shift, known as glutaminolysis, is also observed in freshly resected GSCs (181). Glutaminolysis produces precursors for macromolecules (nucleic acids, amino acids, fatty acids), regulates redox homeostasis (*via* NADH, NADPH, and ROS levels) and contributes to immunosuppression by glutamate production to ensure pro-tumor survival (182). As an abundant non-essential amino acid, glutamine is transported through the blood and capable of crossing the blood-brain barrier, making it a particularly useful energy source by tumors (183).

Glutamate can be converted to α-KG by either glutamate dehydrogenase 1 (GDH1) or transaminases such as glutamate pyruvate transaminase 2 (GPT2) and glutamate oxaloacetate transaminase 2 (GOT2) (184). Conversions fluctuate according to nitrogen and carbon availability. In addition to a metabolite, α-KG behaves as a cofactor (along with oxygen) in the activity of α-ketoglutarate-dependent hydroxylases. These are non-heme, iron-containing enzymes that catalyze a wide range of oxygenation reactions including biosynthesis (ex. collagen and L-carnitine), post-translational modifications (ex. protein hydroxylation), epigenetic regulations (ex. histone and DNA demethylation), as well as sensors of energy metabolism. So far majority of these processes in GBM have been restricted to isocitrate dehydrogenase mutants of GBM, however, collagen prolyl hydroxylases were found to induce metastasis of breast cancer (185) by mechanistically stabilizing HIF-1α in chemoresistance (186). While the importance of HIF-1α in the conversion of GSCs in different tumor niches has been mentioned above, only recently was it reported that collagen-prolyl hydroxylases promote proliferation and invasion in GBM. Interestingly, mouse embryonic stem cells were found to maintain high αKG/succinate ratios *via* glucose and glutamine catabolism that promoted histone/DNA demethylation and maintenance of pluripotency (187). By altering intracellular αKG/succinate ratios, multiple chromatin modifications such as H3K27me3 and ten-eleven translocation-dependent (26) DNA demethylation were shown to regulate genes associated with pluripotency (187). In addition to glucose metabolism, GSCs were observed to have higher expression of genes involved in iron trafficking and metabolism when compared to healthy astroglial and neural progenitor cells, presenting an opportunity for targeted therapeutic intervention (188).

## The Extrinsic Glioma Stem Cell Microenvironment

Unlike the NSC microenvironment in the SVZ, the GBM microenvironment is defined by three unique regions, the hypoxic-necrotic core, the perivascular niche, and the invasive edge, each with distinct contribution to the tumor progression (**Figure 1B**) (189). Each niche influences and activates different cellular programs in GSCs to express distinct markers and transcriptional profiles. This plasticity allows cells to change states and adapt to stressors as needed. The interconnected relationship between GBM and their environment maintains stemness and contributes to heterogeneity which is why emphasis to target the tumor microenvironment has gained traction is recent years, and why more advanced *in vitro* experimental methods such as 3D-culture methods and cerebral organoids are becoming more prevalent (190, 191).

Hypoxia and necrosis are defining features of GBM, caused by the tumor’s exceeding growth requirements on available blood flow to supply oxygen and nutrients. This subsequent lack of oxygen protects cells from irradiation, the most effective treatment modality against GBM, by limiting the amount of molecules capable of turning into cytotoxic free radicals (192). Restricted blood flow also limits the delivery of chemotherapies such as temozolomide to the tumor cells (193). In both cases, the hypoxic environment forces tumor cells into a quiescent state, where the lack of cell division prevents cytotoxic DNA damage induced by chemo-radiotherapies (194). Effects of hypoxia on stemness and tumor survival are largely mediated through hypoxia-inducible factors (HIF-1 and HIF-2), which upregulate signaling pathways including Klf4, Sox2, Oct4, CD133, and VEGF (195, 196). Cell death in the center of the hypoxic region leads to formation of the necrotic core and contributes to the release of pro-inflammatory signals, IL-1β, IL-6, and IL-8, into the surrounding microenvironment. This signaling in turn, contributes to the conversion of tissue-associated macrophages and neutrophils into immune-suppressive and angiogenesis-promoting cells, allowing for continued GBM progression and expansion (197–200). Similar to NSC metabolism, hypoxia forces a metabolic shift in GBM toward aerobic glycolysis and fatty acid metabolism rather than oxidative phosphorylation. Together, the hypoxic niche plays key regulatory roles leading to heterogeneity and cancer progression.

Interestingly, hypoxia can influence GBM cells to transdifferentiate into endothelial-like cells (201) which contribute to feedback loops of the second major tumor environment-the perivascular niche (202, 203). This niche most closely resembles the SVZ where NSCs reside (20, 50, 60). Perivascular niches exist along capillaries or arterioles where endothelial cells come into direct contact with GSCs (204). GSCs in the perivascular niche in turn remodel the microenvironment by producing high levels of pro-angiogenic factors, such as VEGF, that drive endothelial cell proliferation, survival, migration, and blood vessel permeability. This is critical for angiogenesis as GBM is one of the most vascularized human tumors and requires a supply of nutrients for tumor progression. The perivascular niche thus regulates stemness and induces pathways enriching for GSCs, namely nitric oxide and NOTCH (205), TGF-β (206, 207), as well as sonic hedgehog signaling pathways (208). Other perivascular cell populations in this niche, such as tumor-associated macrophages (TAMs) secrete chemokines to promote GSC growth and expansion.

The infiltrating (or “invasive”) edge is the third and final major GBM niche. As a highly invasive tumor, GBMs can infiltrate into healthy brain tissue and limit the effectiveness of maximal surgical resection. To circumvent and eradicate infiltrative GSCs, patients receive whole-brain radiation therapy. However, GBM cells, and most notably GSCs, have been shown to adapt and resist the applied environmental stress of irradiation (85). Once exposed to radiation, cells undergo a process known as the proneural-mesenchymal transition, similar to the metastatic cascade known as the epithelial-mesenchymal transition (EMT). In this process, cells lose cell polarity, cell-cell adhesions, and alter their cytoskeletal organization for migration. GSCs are observed to invade along white matter tracts of the human brain through a NOTCH1-Sox2 mediated feedback loop (209). Mesenchymal GSCs are regulated by STAT3, N-cadherin, NF-κB, and integrins (210–215). These phenotypes exhibited within the infiltrative niche are also influenced by the hypoxic and perivascular niches.

## Leveraging Differences Between Neural Stem Cells and Glioblastoma Stem Cells for Development of Novel Target Therapies

While understanding the similarities between GSCs and NSCs is instrumental for contextualization of gliomagenesis and underlying mechanisms driving GBM progression and therapy resistance, it is leveraging the differences between two cell populations that may offer avenues for generation of novel targeted therapies. Unlike other solid tumors, brain tumors present a unique set of challenges for development of new treatment options. First, the blood-brain-barrier (BBB), which normally protects the brain from harmful toxins, can also hinder the access of targeted therapies against GBM. Although BBB permeability can be theoretically increased through chemical modification of small molecule-based inhibitors, such approach does not expand to more precise modalities including antibody-drug conjugates (ADCs) and adoptive cell transfer therapies. In both mouse models (216, 217) and patient studies, locoregional delivery of the therapeutic offers distinct advantages. In addition to expanding the range of possible treatment modalities, while minimizing systemic toxicities, the locoregional delivery route allows for direct targeting of potential source of GSCs residing in SVZ at the border of lateral ventricle.

One of the most widespread strategies in targeting GSCs is through identification and subsequent development of targeted therapies against cell surface markers. Several different targeting approaches have been investigated in the recent years including antibody-drug conjugates (ADCs) and chimeric antigen receptor T cells (216, 218). And while researchers were able to demonstrate efficacy in mouse models, it is likely that additional combinatorial strategies will be needed to yield complete tumor clearance. Anti-angiogenic therapies have become an attractive modality to prevent tumor progression by cutting off the tumor’s supply of nutrients and oxygen (219). Bevacizumab and other anti-angiogenic therapies showed great promise, but repeated failures show the adaptability of tumors to overcome single agent therapies (220). Reducing the invasion of GBMs has been tested to reduce overall tumor progression, but further therapies would be required to fully eradicate the tumor (221). The use of ibrutinib, and FDA-approved drug to treat lymphoma and leukemia, was shown to suppress the BMX-STAT3 axis in GSCs making them vulnerable to radiation therapy (222). This signaling axis was previously shown to maintain self-renewal in GSCs (223) and mitigate apoptosis (224). Additionally, because BMX is not expressed in neural progenitor cells, ibrutinib may be a selective and beneficial therapy for GBM patients (222).

While there is biological overlap between NSCs and GSCs, promising research is exposing differences and vulnerabilities of each, presenting an avenue for novel therapeutic interventions. Research on telomerase activity in a variety of tumors has resulted in development of distinct therapeutic approaches including small-molecule inhibitors, plant-derived compounds, gene therapy, and immunotherapy. Although they remain to be tested in the clinical setting, several of these therapies have demonstrated promising efficacy in mouse models of GBM (225–228). More recently, CRISPR/Cas9 technology has been tested pre-clinically as a modality to repair mutations in cancers to induce cell cycle arrest with few off-target effects in GBM (229). Moving forward, it will be vital to further interrogate the therapeutic window of telomerase activity modulating treatments by comparing their effects on GSCs and SVZ NSCs. The proximity of SVZ NSCs to the lateral ventricles allows for intracerebroventricular (ICV) delivery of potential therapeutic interventions, bypassing the challenges presented by the BBB while increasing penetrance and distribution. Delivery of cell and gene therapies intracranially and intracerebroventricularly has been tested in both human clinical trials and mouse models of GBM and was shown to reduce tumor progression and invasion (230–232). It is important to note, that due to the extensive intra- and inter-tumoral heterogeneity between GBM tumors, it is unlikely that a single intervention will be sufficient to eradicate the tumor or control its progression, requiring more research into combinatorial approaches in both mouse models and clinical trials. Further research profiling the mechanisms by which SVZ NSCs and the surrounding microenvironment contribute to the chemoradiotherapy evasion of GBM is needed to identify therapies that will synergize with the current SoC. For example, in several *in vitro* and *in vivo* pre-clinical studies, inhibiting CXCL12/CXCR4 signaling in the mouse SVZ promoted radiosensitization and reduced GBM tumor cell proliferation (233, 234). Finally, in the past few years, the difference in the metabolic flexibility between GSCs and NSCs has become more apparent and is now being extensively investigated for the therapeutic potential.

## Concluding Remarks

The aggressive growth characteristic, resistance to therapies and poor clinical outcome have been attributed to the extensive intra- and intertumoral heterogeneity within GBM tumors. Over the years, the observed similarities between GSCs and NSCs of the SVZ, have led to the hypothesis of a transformed NSCs cell presiding at the apex of GBM cytoarchitecture. Although the recent findings have corroborated this hypothesis, it has become evident that that understanding both similarities and differences between GSCs and the healthy NSCs of the SVZ is essential in the search for novel targeted therapies. The comparison of similarities can allow for improved understanding of the molecular mechanisms driving GBM formation, while the comparison of the differences can allow in identifying unique molecular vulnerabilities for development of targeted therapies with a large therapeutic index.

## Author Contributions

All authors (DB, NS, SSa, CV, SSi) contributed to article writing and editing. Figures were generated with BioRender.com. All authors contributed to the article and approved the submitted version.

## Funding

NS is funded by Mitacs Accelerate Fellowship, SSa is generously supported by CIHR CGS-M award and SSi holds Terri Fox Program Project Grant.

## Conflict of Interest

The authors declare that the research was conducted in the absence of any commercial or financial relationships that could be construed as a potential conflict of interest.
